# Replication Stress, DNA Damage, Inflammatory Cytokines and Innate Immune Response

**DOI:** 10.3390/genes11040409

**Published:** 2020-04-09

**Authors:** Sandrine Ragu, Gabriel Matos-Rodrigues, Bernard S. Lopez

**Affiliations:** Institut Cochin, INSERM U1016, UMR 8104 CNRS, Université de Paris, Equipe Labellisée Ligue Contre le Cancer, 24 rue du Faubourg St Jacques, 75014 Paris, France; sandrine.ragu@inserm.fr (S.R.); gabriel-eduardo.de-matos-rodrigues@inserm.fr (G.M.-R.)

**Keywords:** DNA damage response, replicative stress, DNA repair, innate immunity, inflammation, cGAS-STING

## Abstract

Complete and accurate DNA replication is essential to genome stability maintenance during cellular division. However, cells are routinely challenged by endogenous as well as exogenous agents that threaten DNA stability. DNA breaks and the activation of the DNA damage response (DDR) arising from endogenous replication stress have been observed at pre- or early stages of oncogenesis and senescence. Proper detection and signalling of DNA damage are essential for the autonomous cellular response in which the DDR regulates cell cycle progression and controls the repair machinery. In addition to this autonomous cellular response, replicative stress changes the cellular microenvironment, activating the innate immune response that enables the organism to protect itself against the proliferation of damaged cells. Thereby, the recent descriptions of the mechanisms of the pro-inflammatory response activation after replication stress, DNA damage and DDR defects constitute important conceptual novelties. Here, we review the links of replication, DNA damage and DDR defects to innate immunity activation by pro-inflammatory paracrine effects, highlighting the implications for human syndromes and immunotherapies.

## 1. Introduction

The maintenance of genome stability is essential to the accurate transmission of genetic information. Indeed, genome instability is a hallmark of cancer cells [1]. However, the genome is routinely insulted by endogenous as well as exogenous attacks that can generate genetic instability, which ultimately may promote carcinogenesis and/or ageing. Replication and oxidative stresses are major sources of endogenous genotoxic assaults that jeopardize genome stability. Of note, these two endogenous stresses are closely linked, as oxidative stress generates replication stress [2,3]. 

During DNA synthesis, the replication machinery must overcome numerous obstacles, including consequences of oxidative stress, tightly bound DNA-protein complexes, non-B form DNA structures (such as R loop) and lesions that interfere with fork progression [4]. These events can ultimately lead to replication stress by replication fork stalling, collapse or breakage [5,6]. Several mechanisms can be at the origin of replication stress, such as oncogene activation, nucleotide pool imbalance and conflict between replication and transcription [5,6]. These different mechanisms can be connected. Transcription complexes are one prominent endogenous challenge to replication forks. Transcription-replication conflicts (TRCs) can induce DNA replication-fork stalling, DNA recombination, DNA breaks and mutations [7,8,9,10]. Therefore, TRCs pose a potential threat to genome stability [11]. One transcription-associated replication fork barrier is the R-loop, an RNA-DNA hybrid formed when nascent transcripts re-anneal to their template DNA, displacing the non-template strand as single-stranded DNA (ssDNA) [12]. Elevated R-loop levels cause DNA damage and genome instability. The loss of RNA processing and regulatory factors increases R-loop levels, causing R-loop dependent DNA damage in eukaryotic cells [13,14,15,16]. 

DNA breaks and activation of the DNA damage response (DDR), arising from endogenous replication stress, have been observed at early or precancerous stages, and adaptation to replication stress plays an important role in tumour development [17,18,19,20]. The DDR protects genome stability through the precise coordination of a network of pathways, ensuring faithful transmission of genetic material, including DNA replication, repair and recombination, cell cycle checkpoint and chromosome segregation. Ultimately, these autonomous cell responses induce senescence or cell death [21,22,23,24,25]. All these processes prevent the proliferation of cells bearing DNA damage and/or genetic rearrangements. In agreement, syndromes caused by mutations in DDR and/or DNA repair factors are often associated with high genetic instability, cancer predisposition and premature ageing [24,26,27,28]. In addition to these cell-autonomous responses, protective process(es) also act at the organism level. Such mechanism(s) involve the modification of the cellular microenvironment and ultimately the activation of innate immunity.

The inflammatory response is a universal cell-intrinsic response to infections or tissue damage. Inflammation, which is triggered when innate immune cells detect infection, for example, eliminates the initial cause of cell injury, clears out necrotic cells and tissues damaged from the original insult and from the inflammatory process, and initiates tissue repair [29].

To maintain homeostasis, the organisms must maintain a delicate balance in the activation of inflammatory responses. On one hand, an insufficient response results in the susceptibility to infections or tumour development; on the other hand, excessive or self-antigen responses lead to allergy or autoimmune diseases. A possible initial step in innate immune activation is the recognition by the host cell of the foreign features of infectious agents [29,30]. In this case, sensor proteins inspect extracellular and cytoplasmic spaces for the presence of pathogens through pattern recognition receptors (PRRs) and trigger immune responses upon their activation [29,31]. Additionally, the induction of innate immune responses can also occur in the absence of infection, such as when cells recognize self nucleic acids in unwanted subcellular compartments [32].

Pioneering works have shown connections between the immune response and self-DNA and the DDR. It has been demonstrated that DNA damage can trigger innate immune responses through the accumulation of nuclear DNA in the cytoplasm and micronuclei and/or the chronic DNA damage response signalling activation by itself [33,34,35,36]. In this review, we will discuss how replicative stress and DNA damage (mainly DNA double-strand breaks, DSBs) can activate innate immunity and the consequences for autoimmune diseases and immunotherapy.

## 2. Cytoplasmic DNA-Mediated Inflammatory Response

**Figure 1:**
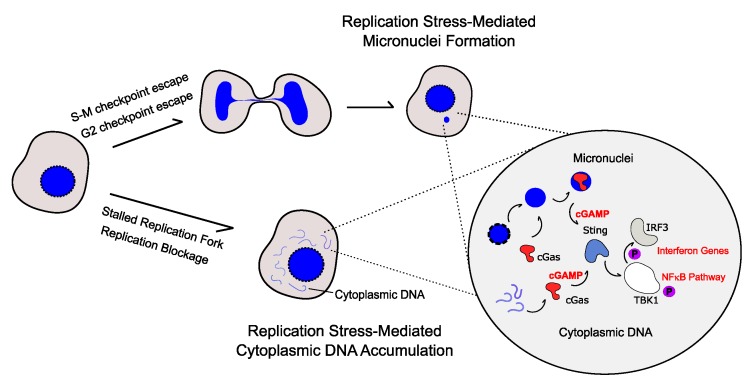
cGAS-STING pathway activation by replicative stress. Two different pathways can trigger cGAS-STING activation by replicative stress: (1) the formation of micronuclei and (2) the accumulation of replication fork-derived DNA in the cytoplasm. These events will trigger the activation of cGAS, which produces cGAMP, activating the STimulator of INterferon Genes (STING) protein. Once activated, STING recruits TBK1 that is then able to phosphorylate its different targets that include Interferon Regulatory Factor 3 (IRF3) and different members of the NF-κB signalling pathway. This culminates in an upregulation of pro-inflammatory factors.

The cyclic GMP-AMP synthase (cGAS)-stimulator of interferon genes (STING), cGAS-STING, pathway plays a pivotal role in the DNA damage-induced innate immune response [34,36,37] (Figure 1). The cGAS protein is a DNA sensor that catalyses the production of cyclic guanosine monophosphate–adenosine monophosphate (c-GMP-AMP or cGAMP) as a second massager [38,39,40,41]. In vitro, biochemical approaches showed that cGAS is able to bind double strand RNA (dsRNA), ssDNA and double strand DNA (dsDNA), but that only dsDNA can drive cGAMP production [42]. Additionally, cGAS is activated in a dsDNA length-dependent manner. Human cGAS is able to bind and be activated by short dsDNA sequences (~20 bp), but longer dsDNA (500–4000 bp) trigger a greater cGAMP production and innate immune activation [42,43,44]. STimulator of INterferon Genes (STING) is a dimeric endoplasmic reticulum (ER)-binding protein that is activated by cGAMP and other forms of cyclic dinucleotides produced by bacterial metabolism [45,46]. The production of cGAMP by cGAS leads to STING oligomerization and translocation to the Golgi apparatus where it activates its downstream targets [47,48]. STING can activate a transcriptional response via the activation of TANK-binding kinase 1 (TBK1) and both the canonical and non-canonical NF-κB pathways [46]. Oligomerization of STING after cGAMP binding creates a signalling platform that recruits and activates TBK1, which reciprocally phosphorylates STING [47,48,49,50]. Phosphorylated STING then binds the transcription factor Interferon Regulatory Factor 3 (IRF3), which is subsequently phosphorylated by TBK1 [47,48,50,51]. Once activated, IRF3 translocates to the nucleus and acts as a transcriptional activator of type I interferon (IFN) genes. Different members of the NF-κB pathway are TBK1 targets. For instance, it has been shown that TBK1 can activate the canonical NF-κB pathway through the phosphorylation of IKB and/or p65 (RelA) [51,52]. In addition, cytoplasmic DNA is able to activate the non-canonical NF-κB pathway in a STING-dependent and TBK1-independent manner by increasing p100 phosphorylation [53,54]. By interacting with its different partners, the cGAS-STING pathway can change the cellular transcriptional program and activate an innate immune response triggered by cytoplasmic DNA.

## 3. Replication Stress Induces the Production of Pro-Inflammatory Cytokines

The accumulation of cytoplasmic ssDNA and dsDNA is also a common feature of tumours and cancer cell lines [34,55,56]. It has been proposed that DNA damage and replication stress elicit the activation of inflammatory responses that contribute to tumourigenesis in some contexts and to senescence/aging in others [57,58]. Indeed, cells exposed to replication stress-inducing agents or deficient in the replication stress response have enhanced production of type I IFNs and pro-inflammatory cytokines that can foster an innate immune response [59,60,61,62].

Two events have been suggested to trigger the activation of the cGAS-STING pathway by nuclear DNA accumulation in the cytoplasm after replication stress (Figure 1). First, the disruption of the replication fork integrity is a major source of cytoplasmic DNA accumulation after replication blockage [59,63]. The inactivation of DNA repair factors that protect and resume arrested replication forks also enhances the accumulation of nuclear DNA into the cytoplasm [63,64]. Importantly, the mechanisms underlying the extrusion of replicating DNA from replication blockage are still not known. As discussed above, in vitro analysis did not show cGAMP production by ssDNA stimulation of cGAS, and some studies reported only the accumulation of ssDNA (single strand DNA) but not double strand DNA (dsDNA) after replication blockage [63,65]. To reconcile these observations, one can propose that secondary structures of the ssDNA can generate the dsDNA sequences able to then activate the cGAS-STING pathway.

Second, upon replication stress, cells containing genomes not fully replicated and/or bearing DNA damage can reach mitosis, leading to mitotic defects, including the formation of micronuclei [66,67,68]. Recent studies have shown that micronuclei structures derived from chromosomal missegregation serve as a platform for the DNA damage-induced immune response [69,70,71,72]. Indeed, exposure to genotoxic chemicals or inactivation of DNA repair factors that leads to replicative stress triggers micronuclei formation and cGAS-STING activation [73,74]. Evidence suggests that micronuclei envelope rupture allows the recognition of the chromatin as cytoplasmic DNA by cGAS-STING [69,70,71].

To prevent aberrant DNA-driven (auto)immune reactions, cells employ a battery of DNases, such as DNase I in the extracellular space, DNase II in endolysosomes or TREX1 (DNase III) in the cytoplasm, that are responsible for the immediate degradation of DNA by-products before the DNA sensing pathways are activated [75]. Different studies have shown enhanced accumulation of replication fork-derived ssDNA in the cytoplasm upon TREX1 inactivation [59,63,64,65,76]. It has been shown that the excess of short free ssDNA that accumulates in TREX1-deficient cells is bound by Replication Protein A (RPA) and RAD51 both in the cytosol and the nucleus [76]. By affecting the availability of proteins such as RAD51, RPA, and MRE11, the innate immune response to replication stress-associated self-DNA may become another determinant of cancer susceptibility to chemotherapy. In summary, these results show that higher eukaryotes cells have several layers of mechanisms to hinder nuclear DNA recognition in the cytoplasm after replicative stress.

## 4. DDR and DNA Repair Defects

Another piece of evidence linking DNA damage and the immune response comes from DDR/DNA repair-deficient patients and animal models. Genetic deficiencies that compromise DDR functions also induce cytokines and lead to auto-inflammatory diseases. Similar to exogenous DNA damage, inherent DNA repair defects in tumours may also increase the production of cytosolic DNA and appear to trigger the STING-dependent response [77].

**Figure 2:**
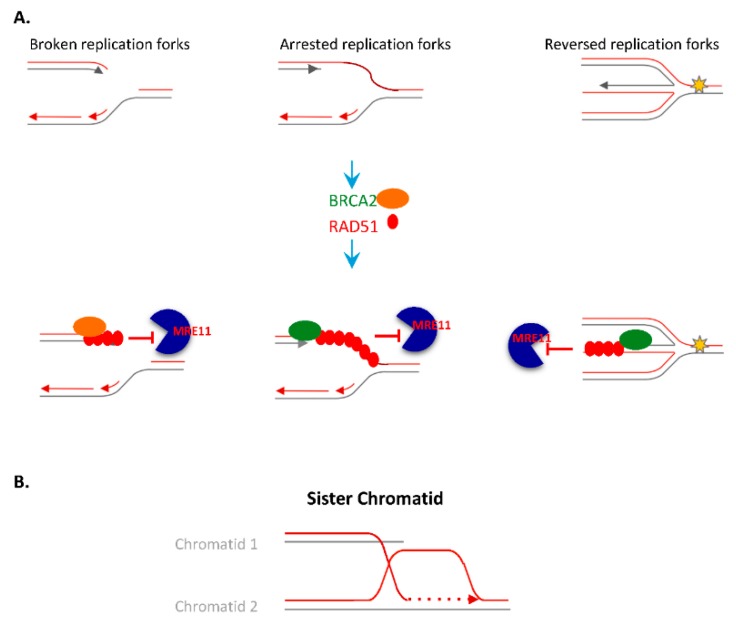
Replication fork protection and restart. (**A**). A replication fork can be broken, or arrested; when reaching an obstacle that arrested it, replication fork can also be reversed generating a so called “chicken foot” structure (right panel). Various actions may take place to protect the replication forks including: BRCA2 loading of RAD51 to protect the forks and/or BRCA2 stabilizing the RAD51 nucleofilament on the single-stranded DNA (ssDNA) regions, thereby preventing MRE11, CtIP, and EXO1-dependent resection (preventing degradation of the arrested forks). (**B**). The strand exchange activity of RAD51 allows then to restart arrested replication forks, using a homologous sequence as matrix, generally the sister chromatid, leading to sister chromatid exchange.

Replication stress is closely related to DNA DSBs. Indeed, the prolonged arrest of replication forks generates DSBs, and, reciprocally, a replication fork reaching a nick or a gap in the copied DNA is converted in a DSB [78] (and for review [6,79]). Additionally, homologous recombination factors such as RAD51 and BRCA2 act to protect the arrested replication fork from degradation, and restarts replication (Figure 2A,B). 

### 4.1. DNA Damage Signalling and Processing of Double-Strand Ends

**Figure 3:**
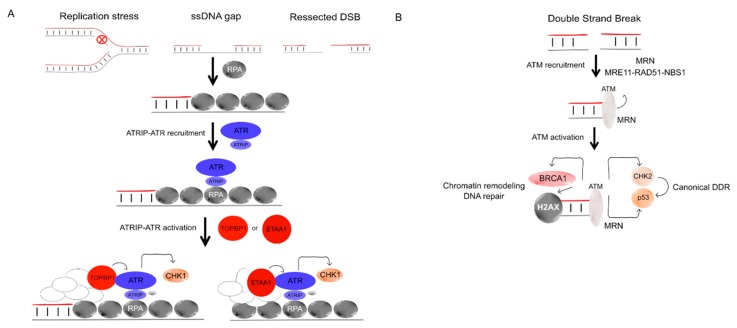
**ATR-interacting protein-ataxia telangiectasia and Rad3 related** (ATRIP-ATR) and ataxia telangiectasia-mutated (ATM) signalling. (**A**). Replication stress events (e.g., replication fork stalling), ssDNA gaps or resected DSB ends lead to the formation of ssDNA stretches. Replication Protein A (RPA) binds the ssDNA stretches and serve as a platform for ATRIP-ATR recruitment. Two modes of ATRIP-ATR activation have been described: in association with accessory proteins TOPBP1 or ETAA1 bind ATR triggering its autophosphorylation. Activated ATR, then, phosphorylate its downstream targets that include the protein kinase CHK1. (**B**)**.** The MRN (MRE11-RAD50-NBS1) complex recognize the DSB. ATM is recruited to the DSB site and binds the MRN complex, which leads to ATM activation. ATM then phosphorylates its downstream targets, including CHK2 and p53 during the canonical DNA damage response (DDR) and/or H2AX and BRCA1 for chromatin remodelling and DNA repair.

DNA damage should first be signalled to coordinate the cell responses, including cell cycle checkpoint and DNA repair/recombination. Two main types of DNA alterations are signalled: ssDNA stretches and DSBs (Figure 3). However, some overlap can exist. Indeed, resection at DSBs generates ssDNA that can then activate the ssDNA-signalling pathways. 

Replication fork blockage and stalling are examples of replicative stress events that leads to the formation of ssDNA stretches [5,80]. Although ssDNA accumulation is the most common origin of replicative stress, ssDNA-independent sources have been reported [5,81]. Replication Protein A (RPA) coated single strand DNA serves as a platform to the recruitment other accessory proteins (e.g., Rad9, ETAA1 and TOBP1) that enable ATRIP-ATR complex activation (Figure 3A). The ATRIP-ATR complex is the central player in the replicative stress response. Along with its downstream target Chk1, these protein kinases phosphorylate hundreds of substrates to control cell cycle arrest, DNA repair and/or cell death [5,82]. Although replicative stress does not refer to the formation of DSB, these events are closely related. The collapse of replication forks can lead to the formation of DSB by replication fork reversion or by endonuclease processing in a process that is accelerated by replicative stress response inactivation [78,83,84]. These DSB ends are recognized by the MRN (MRE11, RAD50 and NBS1) complex (Figure 3B). Once associated with the DSB ends, this complex activates the protein kinase Ataxia Telangiectasia-Mutated (ATM) that is a major signalling hub. ATM targets exhibit a plethora of targets and regulate many aspects of cellular metabolism. Of note, ATM regulates cell cycle and apoptosis through CHK2 and/or p53, for example, or chromatin remodelling and DNA repair by BRCA1 and/or H2AX phosphorylation [85,86]. 

**Figure 4:**
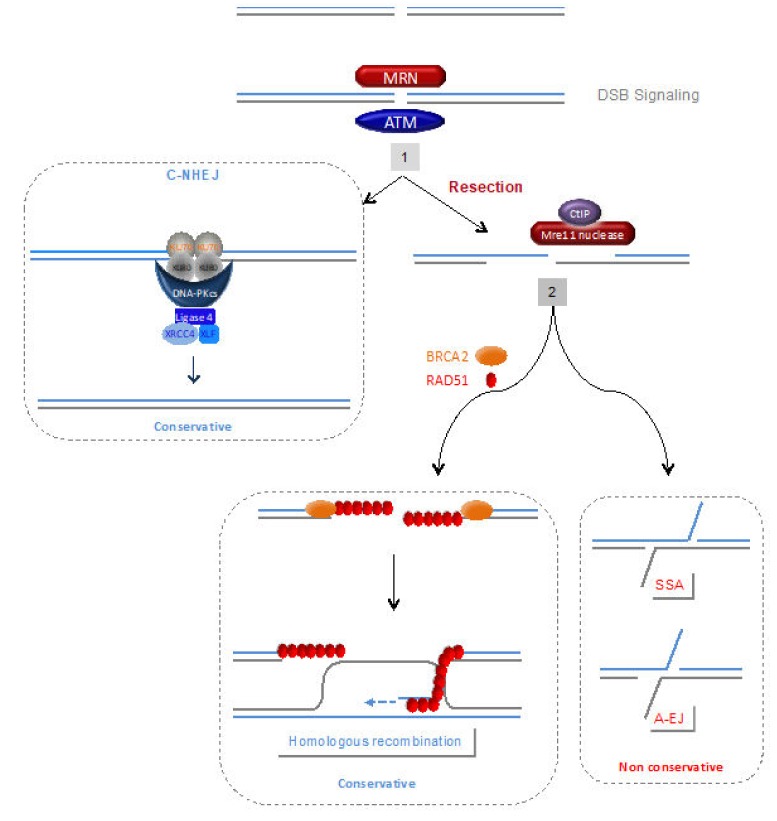
The 2-step DSB repair pathway choice model [79,87,91]. After signalling of the DSB by ATM and MRN, the selection of the DSB repair process act in two successive steps: (1) competition between the canonical NHEJ (C-NHEJ) pathway (KU/DNA-PKcs/ligase 4-dependent) versus resection. Note that C-NHEJ is conservative for DSB repair (for review see [79,87]). The nuclease activity of MRE11 and CtIP favour ssDNA resection, which can then at the second alternative step (2) initiate the conservative homologous recombination (HR) versus non-conservative single-strand annealing (SSA) or alternative end-joining (A-EJ) pathways. Loading of RAD51 on resected ssDNA, by BRCA2, engages DSB repair toward HR. The RAD51 nucleoprotein filament invades the intact homologous duplex DNA, priming DNA synthesis and the intact DNA molecule is copied, creating a D-Loop.

Two general strategies repair DSBs (Figure 4): NHEJ (non-homologous end-joining) ligates the two double strand ends without requiring sequence homologies, while in contrast, homologous recombination (HR) relies on the use of a DNA partner sharing a homologous sequence that provides an intact copy for DSB repair. Alternative DSB repair processes also exist generally based on the use of micro-homologies (2 to 4 bp) at the junctions (For review see [79,87]). HR is frequently proposed to be error-free, while the canonical non-homologous end-joining (C-NHEJ, KU-DNA-PKcs-Ligase 4-dependent) are error-prone processes; however, these are overstatements, and many of the mutagenic end-joining repair events arise in fact from the alternative pathways, as discussed [79,87,88]. Indeed, beside C-NHEJ, highly error-prone alternative(s) end-joining (A-EJ) process(es), also called alt-NHEJ, B-NHEJ (back-up NHEJ), MMEJ (micro-homology mediated end-joining) have been described that inevitably leads to the deletion of the intervening sequence (Figure 4) [87,89,90]. In contrast with C-NHEJ, A-EJ is KU-Ligase 4-independent, but is dependent on PARP1 (for review see [64,65]). Moreover, like HR, A-EJ is initiated by resection of the DSB by MRE11 [91,92,93].

Because, in contrast with C-NHEJ, both HR and alternative end-joining (A-EJ) (and single strand annealing, SSA, see below) are initiated by resection of the DSB generating single strand DNA (ssDNA) we have propose that the choice of the DSB repair pathway acts in two steps: 1- C-NHEJ versus resection; 2- HR versus non-conservative A-EJ or SSA (Figure 4) [79,91,94].

Single strand annealing (SSA) can repair DSB through a homology-mediated process (Figure 4). In this process, the resection of the DSB uncovers two complementary ssDNA tails. The annealing of this two complementary ssDNA repair DSB to the cost of the deletion of the intervener sequence. The annealing step is different from the strand invasion/exchange step performed by RAD51 for HR (see Figure 4 lower left panel); therefore SSA is RAD51-independent [95,96].

HR allows the use of the sister chromatids for the restart of the arrested replication forks. In addition, HR factors, such as RAD51 and BRCA2, protect the arrested replication forks from exonucleolytic single-stranded DNA degradation (resection) initiated by MRE11 [97,98]. C-NHEJ can also seal the DNA ends from collapsed replication forks, reconstituting the continuity of the DNA molecule and thus fostering the resumption of the arrested replication forks. However, on such DNA ends, NHEJ leads to genome rearrangement (for review [79,99]).

Remarkably, the DSB repair processes (mainly C-NHEJ) are critical for the diversity of T and B cells and for an effective adaptive immunity, such as the V(D)J recombination, class-switch recombination and somatic hypermutation (SHM) [100,101]. Of note, R loops have been linked to the regulation of antibody gene diversification [102] and class switch recombination (CSR), which results in the production of the various antibody isotypes that serve crucial effector functions during the humoral immune response [103,104]. R loops form during transcription of switch recombination sequences in vitro and in vivo, and there is solid evidence that R loops are required for efficient class switching. The classical model of R loops posits that they boost mutation rates by generating stable and long tracts of single-stranded DNA that serve as the substrate for activation induced deaminase (AID) [105], the enzyme that initiates the CSR reaction cascade by co-transcriptionally mutating ssDNA in switch recombination sequences [106,107,108,109]. Though logical and compelling, this model has not been supported by in vivo evidence. Indeed, several reports suggest that R loops may not be involved in recruiting AID activity to switch regions, meaning that R loops probably serve other unanticipated roles in CSR. 

Ataxia telangiectasia (A-T), a syndrome associated with impaired DDR, also exhibits pronounced immunodeficiency symptoms [110,111,112]. In other cases, defects in C-NHEJ provoke severe combined immunodeficiency (SCID) [113,114].

In addition to affecting the DSBs’ repair efficiency and accuracy, a defect in the DSB repair pathways also impacts innate immunity activation, as discussed below.

#### 4.1.1. Poly-ADP Ribose Polymerase (PARP1)

The replicative stress response should ensure the identification and signalling of single strand breaks for its processing. PARP1 (a poly-ADP ribose polymerase) plays a multifaceted role in the cellular response to DNA damage, with evidences for involvement in multiple pathways of DNA damage repair and genome maintenance (for review: [115,116]). The existing roles of PARP1 within repair pathways are detection of DNA damage, poly(ADP-ribose) mediated recruitment of repair factors, and in poly(ADP-ribose) mediated regulation of biochemical activities. PARP-1 has now been implicated in maintenance of replication fork stability [117,118,119,120] in the regulation of HR [121,122], C-NHEJ [123,124]), A-EJ, nucleotide excision repair (NER) [125,126], DNA mismatch repair (MMR) [127], and base excision repair (BER) [128,129,130].

It has been shown that inactivation of PARP1 enhances DNA damage and micronuclei-dependent STING activation after replicative stress. In addition to its DNA repair function, PARP1 serves as a transcriptional co-activator in the NF-κB dependent pro-inflammatory transcription response [131]. PARP1 has a direct role in NF-κB-mediated transcription. Indeed, the expression of NF-κB-dependent pro-inflammatory mediators, such as tumor necrosis factor alpha (TNFα), interleukin-6 (IL-6), or inducible nitric oxide synthase (iNOS), is impaired in Parp 1−/− mice [132,133]. Of note, PARP-1 impacts the expression of these NF-κB-dependent pro-inflammatory mediators by inducing the translocation of NF-κB into the nucleus upon genotoxic stress (reviewed in [134]). PARP1 physically interacts with both major subunits of NF-κB (p65 and p50) and is required for NF-κB-dependent gene transcription [132]. PARP1 is also acetylated by the histone acetylase p300/CREB-binding protein (CBP) upon inflammatory stimuli, leading to a stronger association with NF-κB [135]. Hassa et al. also showed that neither the DNA binding nor the enzymatic activity of PARP1 was necessary for the direct transcriptional activation of NF-κB [131]. Therefore, in the context of the replicative stress-mediated inflammatory response, PARP1 exhibits two opposite roles: on one side, by fostering DNA repair PARP1 prevents the activation of the STING-mediated inflammatory response; on the other side, as a co-factor of NF-κB, PARP1 plays a direct active role in the induction the pro-inflammatory transcriptional program. 

#### 4.1.2. ATR and Chk1

The innate immune receptor NKG2D is mostly expressed in NK cells and induces degranulation and cytokine production; thus, contributing to inflammation and NK-mediated cytotoxicity [33]. The recognition of NKG2D ligand (NKG2DL) by the NKG2D receptor greatly contributes to the detection and removal of infected or cancer cells [136]. The DNA damage sensor kinases ATM and ATR, as well as the downstream checkpoint kinase CHK1, were implicated in NKG2DL induction [33]. Ligand upregulation was prevented by pharmacological or genetic inhibition of ATR, ATM or CHK1.

#### 4.1.3. Ataxia Telangiectasia-Mutated (ATM)

ATM is a kinase that plays a key pivotal role in mediating DSB signal transduction for DDR initiation (Figure 3, Figure 4). ATM deficiency causes the ataxia telangiectasia (A-T) syndrome, which is associated with persistent genome instability [137]. The development of a complex multi-organ disease in A-T patients has been proposed to stem from the deregulation of the immune response leading to autoimmunity and chronic inflammation, arising from persistent genome instability in these patients [77]. Fibroblasts from A-T accumulate DNA fragments in the cytoplasm that are recognized by PRRs, inducing an inflammatory response [77,138], which can promote senescence and inhibit stem cell functions [59]. Elevated type I IFN signalling was associated with the ATM deficiency in cultured fibroblasts cell [59,139] and in the sera of human A-T patients and of ATM-null mice [77]. Moreover, ATM−/− mice present with infiltration of neutrophils and lymphocytes in the lungs and increased mRNA levels of pro-inflammatory cytokines, e.g., IL-6 and TNF [140]. Hartlova and collaborators showed that immune activation in A-T patients is driven by damaged DNA in the nucleus that is recognized by the cytosolic innate immune sensor STING [77]. The autoinflammatory phenotype of A-T is abrogated in Atm−/−Sting−/− double knockout mice and significantly reduced after cGAS knockdown, suggesting that the cGAS-STING pathway plays an essential role in the induction of innate immunity upon the accumulation/persistence of DNA damage [77].

#### 4.1.4. The MRN (MRE11/RAD50/NBS1) Complex

The MRN complex is involved both in the early steps of DSB signalling and in the DSB repair mechanism itself, fostering ssDNA resection necessary to initiate HR, as well as alternative DSB repair (Figure 3 and Figure 4) [79,141,142,143,144,145,146].

Hypomorphic NBS alleles exhibit an impaired inflammatory response [147], and a loss of NBS1 leads to exacerbated inflammation [148]. Conditional Nbs1 inactivation in hair follicle (HF) progenitors promotes cytokines secretion and leads to an immune response resembling psoriasiform dermatitis. Additionally, simultaneous Nbs1 and p53 loss, enhance pro-inflammatory cytokines expression and exacerbate this inflammatory phenotype [149]. Moreover, RAD50 interacts with the innate immune system adaptor CARD9 to produce IL-1 [150]. 

Cells with a mutation in the MRE11 gene, derived from a patient with ataxia-telangiectasia-like disorder, and cells in which MRE11 was knocked down had defects in dsDNA-induced type I IFN production. Remarkably, MRE11 physically interacted with dsDNA in the cytoplasm and was required for activation of the stimulator of IFN genes (STING) and IRF3 [151]. RAD50 was also required for dsDNA responses, whereas NBS1 was dispensable. 

MRE11 is also involved in the resection of arrested replication forks, a process counteracted by the HR proteins BRCA2 and RAD51 [97,98]. It has been proposed that MRE11-mediated degradation of the newly replicated genome and the accumulation of these self-DNA fragments in the cytoplasm trigger the activation of STING-mediated innate immune signalling [64]. However, the data observed in Aicardi-Goutières syndrome (AGS), which results from mutation in the SAMHD1 gene, revealed an antagonist role for MRE11, which protects against the production of cytosolic DNA upon replication stress and the subsequent production of interferon [63] (see below). Thus, MRE11 plays 3 roles: it is involved in the signalling of DSBs, in resection initiation and in the signalling of cytosolic DNA.

#### 4.1.5. SAMHD1 and Aicardi-Goutières Syndrome (AGS)

SAMHD1 protects against virus proliferation through its dNTPase activity that degrades nucleotides. A mutation in SAMHD1 can cause AGS [152], an inheritable neurological disease that leads to microcephaly, intellectual disability, and childhood death [153]. It has been proposed that chronic hyper-IFN signalling drives AGS symptoms [154]. Recessive mutations in genes involved in nucleic acid metabolism, such as TREX1, RNASEH2b and SAMHD1 cause AGS [153]. TREX1 (3′ repair exonuclease 1) degrades cytosolic DNA, preventing the induction of cGAS-STING-dependent inflammation [155,156,157,158,159,160]. RNASEH2 degrades RNA/RNA hybrids; thus, avoiding the conflict of replication versus transcription and the resulting replication stress. It should be noted that in RNaseH2-deficient mice, the genetic depletion of STING or cGAS can reverse the ensuing inflammation and autoimmune phenotypes [71,161].

In collaboration with MRE11 and CtIP, SAMHD1 is also involved in resection at endonuclease-induced DSBs and arrested replication forks, i.e., in genomic DNA [60,162]. In SAMHD1-depleted cells, MRE11-dependent resection of stalled replication forks is replaced by the RecQ1 helicase unwinding of the DNA; then, by cleaving the DNA flap generated, an endonuclease generates ssDNA fragments that accumulate in the cytosol, where they activate the cGAS-STING pathway, inducing the expression of pro-inflammatory type I IFN. By favouring MRE11 activity at arrested replication forks (instead of RecQ1), SAMHD1 thus protects against such a response, preventing chronic inflammation by limiting the release of ssDNA from stalled replication forks [60].

### 4.2. Homologous Recombination (HR)

RAD51 plays a pivotal role at the central step of HR, i.e., the search for homology and strand exchange. RAD51 is loaded on resected ssDNA by BRCA2 (Figure 4). Moreover, in addition to promoting the search for homology and strand exchange, RAD51 and BRCA2 protect arrested replication forks from extensive degradation by MRE11/SAMHD1 (Figure 4). Then, RAD51 helps the restart of the arrested replication forks using the sister chromatid as a template (Figure 2B). RAD51 thus plays a pivotal role in genome plasticity. It is noteworthy that BRCA1, which elicits the ssDNA resection and thus HR initiation, and BRCA2 are frequently mutated in familial ovary and breast cancer [163,164]. Many genes directly or indirectly controlling HR are mutated in Fanconi anaemia (FA) syndrome, which is associated with developmental defects, genetic instability, bone marrow failure and cancer predisposition. 

Inhibiting RAD51 functions leads to replication stress, which then generates mitosis defects, uneven chromosome segregation and micronuclei formation [68,165]. Moreover, in the absence of RAD51, the unprotected newly replicated sequences are degraded by the exonuclease activity of MRE11, and the fragmented nascent DNA accumulates in the cytosol, initiating triggering the activation of STING-mediated innate immune signalling [64].

BRCA2 inactivation also leads to pro-inflammatory cytokine production, including TNFα, and increases sensitivity to TNFα. Enhanced TNFα sensitivity is also present in BRCA1 or FANCD2 inactivation [166]. BRCA2 inactivation leads to cGAS-positive micronuclei and results in a cell-intrinsic interferon response (cGAS/STING-mediated interferon response), which encompasses rewired TNFα signalling and enhances TNFα sensitivity [166].

Defects in BRCA1 result in the constitutive activation of the STING pathway in response to accumulation of cytosolic DNA [49,167]. Additionally, BRCA1 plays a hitherto unidentified role as a cofactor to IFI16 in the nuclear innate sensing of foreign DNA and the subsequent assembly and cytoplasmic distribution of stable IFI16-inflammasomes leading to IL-1β formation, as well as the induction of IFN-β via cytoplasmic signalling through IFI16, STING, TBK1 and IRF3 [168]. 

#### 4.2.1. Resolution of HR and Arrested Replication Forks Intermediates

Reversion and resumption of arrested replication forks, and HR intermediates (Holliday junctions), generate branched DNA structures that are resolved by cleavage or dissolution [6,67,79]. A defect in these activities leads to accumulation of such branched structures that are toxic and can generate genetic instability [169].

##### a—SLX4 and MUS81

SLX4 is a platform protein that recruits nucleases that resolve branched DNA intermediates resulting from HR and/or arrested replication fork processing. MUS81 is an endonuclease is an endonuclease that interacts with SLX4 and plays a role in resolving HR intermediates, suppressing chromosomal instability [170]. MUS81 fosters the accumulation of fragmented self-DNA (including DNA lesions, R-loops, repetitive sequences and common fragile sites) resulting from replication stress, leading to STING-dependent expression of type I IFNs and chemokines [171]. SLX4 is mutated in FA Group P. Fibroblasts from FA-P harbour cytoplasmic DNA accumulation, including sequences deriving from active Long INterspersed Element-1 (LINE-1), triggering the cGAS-STING pathway that elicits IFN expression. Similar results were obtained with FA-D2, an upstream activator of SLX4, which caused the accumulation of DNA fragments in the cytoplasm that are recognized by PRRs, inducing an inflammatory response [138] leading to senescence and inhibiting stem cell function [58]. In vitro, stimulated FA bone marrow showed elevated levels of tumour necrosis factor-α (TNF-α) and IFN-γ [172]. Sumpter et al. suggest that FA genes function in the selective autophagy of genetically distinct viruses, in mitochondrial quality control and in preventing inflammasome activation due to mitochondrial reactive oxygen species (ROS) [173].

##### b—RecQ helicases

The RecQ helicase family contains five members, including RecQ1, RecQ2, RecQ4 (mutated in Rothmund–Thomson syndrome), Bloom syndrome (BLM) and Werner syndrome (WRN). Many of these helicases are involved in the processing of HR and replication stress intermediates.

As discussed above, the ablation of SAMHD1 decreases MRE11-mediated resection of arrested replication forks; the resection at blocked forks is then performed by RecQ1, which unpairs the two DNA strands, after which an endonuclease that cleaves the displaced ssDNA strand then produces a piece of DNA that can be exported to the cytoplasm, triggering the production of IFN1 through the STING pathway [63].

BLM is deficient in Bloom syndrome (BS), a rare genetic disease characterized by genome instability, increased sister chromatids exchanges, accumulation of micronuclei, susceptibility to cancer and immunodeficiency [174]. BLM-deficient fibroblasts show constitutive upregulation of inflammatory interferon-stimulated gene (ISG) expression, which is mediated by the cGAS-STING-IRF3 cytosolic DNA-sensing pathway [73]. Moreover, increased DNA damage or downregulation of the cytoplasmic exonuclease TREX1 enhances ISG expression in BLM-deficient fibroblasts, and BS patients show elevated ISG expression in the peripheral blood [73]. BLM, in association with Dna2 and EXO1, has been reported to promote long-range ssDNA resection [175,176]. In contrast with MRE11 and CtIP, depletion of these factors suppressed the increase in signal of short cytoplasmic ssDNA detected in cells treated with ionizing radiation (IR), mitomycin C or cisplatin, which activated the innate immune response, and TREX1 deficiency exacerbated these responses [65]. The absence of involvement of MRE11 and CtIP may suggest that only long-range resection produces reactive ssDNA species. Moreover, in contrast, BLM has also been reported to protect against ssDNA resection through the loading of 53BP1 on DSBs in the G1 phase but not in the S/G2 phase [177]. Collectively, these data suggest that the role of BLM in generating cytosolic DNA acts in the S phase, thus as a consequence of replication stress. 

A deficiency in WRN leads to Werner syndrome (WS), which is associated with genetic instability, cancer predisposition and premature ageing [178,179,180]. Fibroblasts derived from WS patients or from mutant mice and serum from WS patients show increased inflammatory signalling characterized by expression changes in HIF-1, IL-6 and components of the NF-κB pathway [181,182,183].

### 4.3. C-NHEJ

DNA-dependent protein kinase (DNA-PK) is formed by the association of the heterodimer KU70-KU80 with DNA-PKcs (DNA-PK catalytic subunit). DNA-PKcs is the catalytic subunit of the kinase that plays a central role in C-NHEJ regulation (Figure 4). Germline mutations affecting DNA-PKcs lead to severe combined immunodeficiency (SCID) [184]. Indeed, DNA-PKcs is required for V(D)J recombination, which utilizes the NHEJ pathway to promote antigen diversity in the adaptive immune system. Additionally, the Activation Induced Cytidine Deaminase (AID) also helps in generation of immunoglobulin diversity by mediating somatic hypermutation and gene conversion [185].

DNA-PKcs interacts with DNA in the cytoplasm and is critical for inducing innate immune responses to DNA and viral DNA in fibroblasts [186,187,188]. A novel role for DNA-PK in activating innate immunity and the inflammatory response independently of NF-κB has been, thus, suggested [186]: the DNA from various pathogens is bound by DNA-PK, resulting in IRF-3-mediated transcription of multiple cytokine and chemokine genes independently of DNA-PKcs kinase activity [186]. Furthermore, evidence shows that KU70 induces IFN-λ1 production by activating the IFN regulatory factors IRF-1 and IRF-7 in response to cytosolic DNA [188,189]. 

### 4.4. Other DNA Repair Pathways 

The excision repair pathways, nucleotide excision repair (NER), base excision repair (BER) and mismatch repair (MMR) repair a variety of DNA lesions. NER is specific for the repair of bulky adduct DNA damage [190,191,192], while BER is responsible for removing damaged bases containing small oxidative and alkyl adducts [193,194,195,196,197]. The MMR pathway is capable of repairing single base mismatches and a variety of small insertions and deletions to the genome [198,199]. Chronic innate immune activity was detected and found to cause tissue deterioration in NER-deficient ERCC1 mutant mice [200,201]. Immunological symptoms are also present in mice lacking OGG1, a DNA glycosylase playing a pivotal role in BER [202,203] and in APE2-deficient mice (AP endonuclease involved in BER) [204]. Moreover, another BER glycosylase, the apurinic/apyrimidinic endonuclease 1 (APE1), has been associated with the activation of immune signalling [205]. In a preclinical murine model, the isogenic introduction of a mutation in the central MMR player MSH2 (MutS homologue 2) resulted in marked improvements in the immune response [206,207].

## 5. DDR and Autoimmune Disease

Autoantibodies against the KU antigen, which were originally described in patients with scleroderma-polymyositis overlap syndrome, are also found in many autoimmune diseases, particularly in patients with systemic lupus erythematosus (SLE), systemic sclerosis (SSc), and undifferentiated connective tissue disease (UCTD) [208]. Autoantibodies against KU, DNA-PKcs, poly-ADP ribose polymerase (PARP), and against DNA repair proteins such as WRN and MRE11 have been identified in the serum of patients having systemic autoimmune rheumatic disease (SARD) [209]. Another study reflecting the role of DNA damage in promoting autoimmune diseases showed that cell lines from patients with systemic lupus erythematosus (SLE) have a defective DSB repair [210]. In the same context, Bawadekar et al. (2015) have shown the presence of IFI16 in the sera of systemic-autoimmune patients associated with an upregulation of cytokine-encoding genes in endotoxin-free recombinant IFI16 (rIFI16) endothelial cells. IFI16 seemed to promote inflammation in endothelial cells through the activation of p38 MAPK and NF-κB p65 [211]. Finally, Karakasilioti et al. (2013) showed that persistent DNA damage signalling in mice, carrying a defective NER only in adipose tissues, triggers a chronic autoinflammatory response leading to fat depletion and metabolic abnormalities [200].

## 6. Reactive Oxygen Species and Nitrogen Species

Reactive oxygen species (ROS) comprise of a family of short-lived molecules, such as O_2_^−^, H_2_O_2_ and •OH [212]. ROS can potentially alter every kind of biological molecules. For example, ROS can generate DNA damage and can affect replication dynamics through different processes, including DNA damage that impairs polymerase progression, imbalance of the nucleotide pools (through oxidation of the ribonucleotide reductase), oxidation of proteins of the replisome. Nitrogen species (RNS) can also damage DNA to form mutagenic lesions, 8-nitroguanine [2,213,214,215,216].

On the other hand, ROS can exercise active roles as secondary messengers in physiological processes [217]. Many functional roles of ROS have been shown in cells from aiding immunity (e.g., oxidative bursts in phagocytes to eliminate pathogens) [218] to acting as signalling molecules (e.g., H_2_O_2_ regulating NFκB, MAPK pathways) [219]. 

ROS are produced endogenously by mitochondria (where O_2_ acts as a terminal electron acceptor for electron transport chain) [220], NADPH oxidase, a cell membrane bound enzyme [221], peroxisomes (which contain enzymes that produce H_2_O_2_ e.g., polyamine oxidase) [222], endoplasmic reticulum (produce H_2_O_2_ as a by-product during protein folding); or upon exposure to exogenous stress like ionizing radiation (IR), chemotherapeutic drugs and environmental insults, which affect the organelles and enzymes listed above [223]. Depending on the source of ROS, cell type, and tissue environment, ROS signalling may participate in normal physiological processes or contribute to a maladaptive response that leads to metabolic dysfunction and inflammatory signalling. ROS can generate cytosolic DNA, which is sensed by cGAS within tumours. The cGAS, in turn, activates STING to upregulate the expression of type 1 IFN, ISG and SASP genes [224]. The oxidized base 8-hydroxyguanosine (8-OHG), a marker of oxidative damage in DNA, potentiated cytosolic immune recognition by decreasing its susceptibility to repair exonuclease1 (TREX1)-mediated degradation. Thus, oxidized DNA represents a prototypic damage-associated molecular pattern (DAMP) with important implications for infection, sterile inflammation and autoimmunity [225]. Mitochondria-derived ROS can induce activation of innate immune responses, including activation of inflammasome, cGAS-STING and NF-κB signalling pathways (for review: [226]). The ambient levels of ROS are important for homeostasis of cells, whereas excessive ROS are important in killing pathogens. If ROS are not controlled by an array of sophisticated antioxidant mechanisms, it leads to inflammatory tissue injury [227].

### A Vicious Circle

Under inflammation, ROS and RNS are produced in inflammatory and epithelial cells causing damages to a wide variety of biomolecules including nucleic acids, proteins and lipids [228,229]. Under inflammatory conditions, nitric oxide (NO) is generated in inflammatory and epithelial cells, and this reaction is catalysed by NO synthase (NOS), especially inducible nitric oxide synthase (iNOS). NO and NOS are known to play roles on both pro- and anti-carcinogenic effects [230]. Sustained induction of iNOS in chronic inflammation can produce ROS and RNS, causing DNA damage [231].

## 7. Implications for Immunotherapy 

Alterations in DNA repair and replicative stress can influence either the adaptive immune system, by increasing the number of mutations/neoantigens leading to increases in the foreignness of a tumour/antigenicity [232], or both the innate and adaptive immune systems, by changing the cell microenvironment. Neoantigens result from mutations and encode immunologically active proteins that can cause the immune system to recognize the affected cell as foreign. Defects in DNA repair may influence the adaptive immune system by leading to an increased number of mutations and subsequently to an increased number of neoantigens, which in turn increases the foreignness of a tumour (i.e., antigenicity), resulting in a higher probability of tumour recognition by the immune system. DNA repair could also influence how the innate immune system initially responds to a tumour and recruits the adaptive immune system to the site of malignancy. DNA repair deficiency is associated with upregulation of immune checkpoints and immune cell-infiltrated microenvironments. While activation of immune pathways such as STING in the acute phase promotes an antitumourigenic response, in the chronic phase, DNA damage repair-deficient tumours instead exploit this STING-mediated immune response, tailoring it to promote a proinvasive microenvironment favouring tumour growth. Indeed, defects in DDR improve the recognition of tumours by the adaptive immune system [233]. Before the adaptive immune system can recognize a tumour as foreign, immune cells must be recruited to the site of the tumour. 

In vivo studies revealed that chemotherapy [234] and ionizing radiation treatment [235,236,237] induce type I IFN signalling in tumours to promote antitumour immunity. Burdette et al. suggested that this recognition is often mediated by STING activation [45], highlighting the important role that the cGAS-STING pathway plays in innate immune response activation during tumour progression. Even though cGAS-STING has pro- and antitumourigenic roles, depending on the context [35,46,238], this pathway has been suggested as necessary for a therapeutic response in immunotherapy. Studies performed in a syngeneic MC38 murine tumour model showed that the knockout of STING significantly reduced tumour control after single-dose ionizing radiation [237]. Similar to exogenous DNA damage, inherent DNA repair defects in tumours may also increase the production of cytosolic DNA and appear to similarly trigger a cGAS-STING response [77]. Indeed, blocking T cell inhibitory pathways using immune checkpoint inhibitors is ineffective in mice lacking STING [69]. The ability of transformed cells to inactivate this pathway is supposed to create a selective advantage, as cGAS-STING signalling is suppressed in several tumours [239].

In opposition, the cGAS-STING pathway has pro-tumorigenic roles in contexts of inflammation -induced tumorigenesis [238]. For example, it has been shown that genetic inactivation of *Sting* in mice has an anti-tumorigenic effect in skin cancer induced by 7,12-dimethylbenz[a]anthracene (DMBA) [240] and colorectal cancer induced by azoxymethane (AOM) [241]. Additionally, STING can also participate on tumour progression. In a model of breast cancer, the activation of IFN-STAT1 signalling by STING enhanced cell survival and increased the resistance to DNA damage induced chemotherapy [242]. These results highlight the importance of context specificity to the use of STING inhibitors for cancer therapy.

In ovarian cancers, BRCA1/2-mutated tumours are associated with increased levels of tumour-infiltrating lymphocytes [243]. These patients show improved prognosis, and in addition to increased genomic instability, these tumours have more frequent STING activation [243]. In the same context, inactivation of replicative stress response factors (PARP1 and/or ATR inhibition) enhances the cGAS-STING-mediated interferon response after BRCA2 inactivation in human cell lines [74,244]. Similar results have been shown in small cell lung cancer (SCLC) after the inhibition of either PARP1 or CHK1 [245,246]. 

A synergic relationship between the inactivation of replicative stress response inhibitors and replicative stress factors has been shown in clinical trials [247,248]. The new findings on the interaction of replicative stress and the innate immune response provide exciting novelties that can impact the development of new therapeutic strategies for cancer.

## 8. Conclusions

Cells and organisms are inevitably subjected to exogenous and endogenous stresses that jeopardize genome integrity. Several levels of responses have been developed to face such stresses. At the cellular level, the DDR, programmed cell death and senescence programmes avoid the proliferation of cells bearing DNA damage and rearrangements. However, the second level of defences, at the organismal level, has emerged from recent studies: the activation of innate immunity by DNA injuries, thus allowing for the elimination of cells bearing DNA damage.

Indeed, an exciting new concept is the link between replication stress and the activation of the cell-intrinsic innate immune response. Many findings reveal that exposure to agents generating replication stress and replication stress-deficient cells engender the expression of pro-inflammatory cytokines and type I IFNs. This activation is mediated through the presence of cytosolic DNA. Remarkably, although this DNA corresponds to genomic DNA from the cell, it is recognized as a foreign DNA by the defence systems. Central to the innate immune response is the adaptor protein STING, which couples signals from cytosolic DNA sensors to a transcriptional response for the activation of type I IFN signalling axes, promoting elimination by the adaptive immune system. STING signalling is suppressed in several tumours, and multiple cancer cell types contain genome-derived cytosolic ssDNA, affirming the presence and importance of persistent replication stress in tumours. As type I IFN production from the innate response is critical in priming the adaptive immune system, robust STING signalling has been associated with an increased immunotherapy response. Future studies should allow for a better understanding of the interplay between replicative stress and the immune system and should provide insight into how these responses can be regulated optimally. This knowledge might also allow for the improvement of anticancer strategies connecting radio-/chemotherapies with immune therapy.
